# Predicting spatial and temporal variability in crop yields: an inter-comparison of machine learning, regression and process-based models

**DOI:** 10.1088/1748-9326/ab7b24

**Published:** 2020-02-28

**Authors:** Guoyong Leng, Jim W. Hall

**Affiliations:** 1 Key Laboratory of Water Cycle and Related Land Surface Processes, Institute of Geographic Sciences and Natural Resources Research, Chinese Academy of Sciences, Beijing 100101, China; 2 Environmental Change Institute, University of Oxford, Oxford OX1 3QY, UK

**Keywords:** climate change, crop yield, machine learning, statistical model, crop model

## Abstract

Pervious assessments of crop yield response to climate change are mainly aided with either process-based models or statistical models, with a focus on predicting the changes in average yields, whilst there is growing interest in yield variability and extremes. In this study, we simulate US maize yield using process-based models, traditional regression model and a machine-learning algorithm, and importantly, identify the weakness and strength of each method in simulating the average, variability and extremes of maize yield across the country. We show that both regression and machine learning models can well reproduce the observed pattern of yield averages, while large bias is found for process-based crop models even fed with harmonized parameters. As for the probability distribution of yields, machine learning shows the best skill, followed by regression model and process-based models. For the country as a whole, machine learning can explain 93% of observed yield variability, followed by regression model (51%) and process-based models (42%). Based on the improved capability of the machine learning algorithm, we estimate that US maize yield is projected to decrease by 13.5% under the 2°C global warming scenario (by ~2050s). Yields less than or equal to the 10^th^ percentile in the yield distribution for the baseline period are predicted to occur in 19% and 25% of years in 1.5°C (by ~2040s) and 2°C global warming scenarios, with potentially significant implications for food supply, prices and trade. The machine learning and regression methods are computationally much more efficient than process-based models, making it feasible to do probabilistic risk analysis of climate impacts on crop production for a wide range of future scenarios.

## 1 Introduction

Understanding climate impacts on crop yields is fundamental for enhancing agricultural resilience in a changing climate. Numerous studies have been conducted for assessing the impacts of climate change on crop yield at the regional and global scales (Olesen and Bindi 2002; Jones and Thornton 2003; Parry *et al* 2004; Tao *et al* 2006; Yao *et al* 2007; Schlenker and Roberts 2009; Reidsma *et al* 2010; Schlenker and Lobell 2010; Bindi and Olesen 2011; Müller *et al* 2011; Urban *et al* 2012; Rosenzweig *et al* 2014). In general, high temperature exerts negative impact on yields directly through heat stress and indirectly via soil moisture deficits, while low precipitation tends to induce closure of stoma, reduction of carbon uptake and decrease of yields (Peng *et al* 2004; Schlenker and Roberts 2009; Butler and Huybers 2013; Asseng *et al* 2015; Ray *et al* 2015; Liu *et al* 2016; Zhao *et al* 2016; Schauberger *et al* 2017; Wang *et al* 2017).

Despite tremendous efforts to quantify yield response to climate variation, substantial uncertainties still exist in the estimation of climate impacts on crop yield (Asseng *et al* 2013; Wheeler and von Braun 2013; Challinor *et al* 2014b; Wang *et al* 2017), which can be attributed to the complexity of the relevant processes and the scarcity of relevant datasets. In general, two types of models have been widely used in the literature: statistical models (Lobell and Field 2007; Tebaldi and Lobell 2008; Zampieri *et al* 2017; Gaupp *et al* 2019; Leng and Hall 2019) and process-based models (Rosenzweig *et al* 2014; Waha *et al* 2015; Deryng *et al* 2016; Müller *et al* 2017). Whilst each approach has its own strengths and weakness, only a few studies have compared the estimates between statistical models and crop models (Leng 2017a, b; Lobell and Asseng 2017; Roberts *et al* 2017).

For the most part, the focus of these studies, and inter-comparison exercises such as the Agricultural Modelling Inter-comparison and Improvement Project (AgMIP; (Rosenzweig *et al* 2013) has been on understanding the potential future changes in average agricultural yields given future climate scenarios. There has been much less attention to the impacts of climate variation and extremes, though these effects can be particularly significant for global food systems if they result in large reductions in yield, interruptions to supply and price increases (Gaupp *et al* 2018). Empirically characterizing the variability in crop yields is more challenging than characterizing general trends, in particular because extreme events are by definition rare. Furthermore, using crop models to characterize variability in time involves many computationally expensive simulations, whilst simulating variation in space requires high resolution datasets that may not be available.

Some more studies have recently examined the effects of droughts on agricultural production (Leng and Hall 2019) and the impacts of concurrent droughts in different locations (Gaupp *et al* 2017, 2019). Although these studies have addressed large food producing regions, they have not sought to characterize both the spatial and temporal response of yields to climatic variability. Better resolution of yield variations in space and time is needed in order to understand the risks of climatic variability for agricultural productions. Here, our hypothesis is that neither established crop models or conventional regression models are well-suited to this task. Crop models have been mainly used for understanding and simulating trends in crop yields and agricultural production (Elliott *et al* 2014; Rosenzweig *et al* 2014; Zhao *et al* 2017), in order to inform policy decisions, whilst regression models are traditionally linear, which may not be appropriate given the complex response of crop yields to external perturbations.

Recently, machine-learning (ML) has emerged as a powerful tool for environmental analysis (Chlingaryan *et al* 2018), as well as climate impact assessment on crop yield (Jeong *et al* 2016; Johnson *et al* 2016; Feng *et al* 2018; Cai *et al* 2019; Feng *et al* 2019; Vogel *et al* 2019). ML often shows better performance compared to conventional linear regression models (Feng *et al* 2018), as it can capture non-linear relationships, handle the interactions among predictors and do not assume a certain shape of response function (e.g. linear or polynomial) (Breiman 2001; Shalev-Shwartz and Ben-David 2014). We hypothesize that these characteristics of ML will enable better prediction of variability of crop yields in space and time. However, a comprehensive inter-comparison of process-based crop models, regression and ML is lacking, especially for prediction of variation and extremes in crop yields in relation to spatially and temporally varying climatic effects.

In this study, we choose the US maize yield as an example to demonstrate the potential utilization of ML for assessing climate impacts on crop yields, through comparing the simulations by process-based models, regression and ML in a consistent manner. Specifically, the following scientific questions are addressed in this study: 1) How ML performs in assessing climate impacts on maize yields in the United States? 2) How large are the uncertainties arising from climate impact assessment models? 3) How the average, variability and extremes of US maize yield would change in the future under global warming scenarios? Through a county-level analysis, we aim to identify where maize yield estimation is most uncertain and where assessment of yield response to climate change is relatively more promising, thus providing critical information for climate risk assessment and effective adaptations.

## 2 Materials and Methods

### 2.1 Crop yields and climate data

Maize yield census data is obtained from the US Department of Agriculture (USDA)’s National Agriculture Statistics Survey’s (NASS) Quick Stats database (http://www.nass.usda.gov/Quick_Stats). Simulated maize yields by 11 process-based models (Supplementary Table S1) are obtained from the Agricultural Modelling Inter-comparison and Improvement Project (AgMIP) (Rosenzweig *et al* 2013) and the Inter-Sectoral Impact Model Inter-comparison Project (ISI-MIP) (Warszawski *et al* 2014). Some of the 11 process models were calibrated with regard to certain parameters (Supplementary Table S1). For example, the parameter maximum LAI, harvest index and the factor for scaling leaf-level photosynthesis to stand level are calibrated in the LPJmL model, while the parameter fertilizer application rate and potential harvest index are calibrated in the GEPIC model. More details on the process models can refer to (Müller et al., 2017). The crop models have been run under three different configurations (Elliott *et al* 2015). The default setup represents each modelling group’s own assumptions, while the key input data, parameters and definitions provided by AgMIP are adopted by the models under the setting of “fullharm”. Models that consider nitrogen are also run without nitrogen stress under the “harmnon” setting. Five climate model simulations (Supplementary Table S2) from the Coupled Model Intercomparison Project Phase 5 (CMIP5) (Taylor *et al* 2012) under Representative Concentration Pathways RCP8.5 (Moss *et al* 2010) are used to drive crop models for simulating future yield changes. These climate model projections are statistically downscaled to 1/8 degree and bias-corrected against observations using bias-correction and spatial-downscaling approach (BCSD) (Wood *et al* 2004; Leng *et al* 2016). The historical period 1980-2010 is selected because it overlaps with both census yield data and process-based model simulations.

### 2.2 Machine Learning and regression model

Besides process-based crop models, we use ML and a traditional multiple linear regression model to simulate maize yield. Here, the Random Forest algorithm (Breiman 2001) which has been successfully used in previous studies (Hoffman *et al* 2018; Feng *et al* 2019; Vogel *et al* 2019) is adopted. The Random Forest algorithm is a non-parametric ML method and relies on an ensemble of decision trees through two randomization steps: 1) each decision tree is constructed based on a bootstrapped sub-sample dataset, with the decision rule depending on a random subset of candidate predictor variables; 2) These processes are repeated at every decision split to overcome the limitations of single decision tree, thus avoiding the potential overfitting issue (Breiman 2001). Compared to traditional statistical approaches (e.g. multiple linear regression), Random Forests are expected to have a number of advantages (Cutler et al 2007), as they do not assume a certain shape of response function (e.g. linear or polynomial). That is, the complex interactions and nonlinear relations among the variables could be handled.

Following previous studies (Lobell and Asner 2003; Lobell *et al* 2011; Leng 2017a, b), two climate variables and the year term are selected in this study as inputs for both the ML and regression models. That is, the growing season (June-July-August) temperature and precipitation are selected to represent the first-order climate impacts on yields, while the change trend of yield is accounted for by the year term in the model. Climatic extremes are not explicitly considered in this study, since it is more difficult for CMIP5 climate models to predict future changes in climate extremes than climatology. Specifically, the multiple linear regression model is constructed with temperature (T), precipitation (P) and year (Y) as the predictant and maize yield (MY) as the dependent variable:

(1) $$MY_{c,y}=\beta_{0}+\beta_{1}T_{c,y}+\beta_{2}P_{c,y}+Y_{y}+\alpha_{s}+\varepsilon_{s,y}$$

Where *β*_0−2_are the intercept and coefficients, *c* is for the county and *y* is the year, *α_s_*is the intercept representing constant regional effects, *ε_c,y_* denotes the error term. The same time series of three predictors are also used as inputs into the ML model, which is run under the default settings with ntree (the number of trees to grow in the forest) set to be 500 and mtry (the number of randomly selected predictor variables at each node) being 2. Here, a “complete” model refers to the one that includes both climatic factors governing yield variability and the year term representing the change trend of yields.

### 2.3 Simulation and Analysis

In this study, eighty percent of the dataset was randomly selected to calibrate both the ML and regression model, with the remaining data used for validations. This procedure was executed 100 times with different randomly selected calibration and validation datasets to evaluate model stability. The “complete” models which include both climatic factors and the year term (see equation 1) are developed for simulating historical maize yields, based on which inter-comparisons of the long-term mean and year-to-year variation of maize yield are conducted among process-based models, regression model and ML. To assess the portion of yield variability that is contributed by climate variations, a “reduced” model which includes climate variables only is constructed. Specifically, both climate variables and yields are detrended using the least squares linear regression method, before they are used for fitting the “reduced” models. The simulated yield anomaly is then compared with observed yield anomaly to evaluate the variance of yield anomalies that can be explained by climate variations for each maize growing county. Country-level values are obtained based on aggregated yield anomalies through weighted arithmetic mean with harvested area as weighting factors. This calculation ensures that grid cells with large harvesting areas have greater impacts on regional yield anomalies.

### 2.4 Projection of yield change under the 1.5°C and 2°C warming scenarios

Most previous studies have focused on how crop yields will change on average in a changing climate, with less emphasis on predicting potential changes in low yields as a consequence of climate variations. To illustrate the usefulness of ML method for this purpose, we examine how climate change could impact upon the likelihood of low yields defined as the historical 10^th^ percentile yield. Instead of looking at the far future periods such as the end of 21st century, we limit our predictions to the 1.5°C and 2°C warming scenarios (relative to pre-industrial levels) (Rogelj *et al* 2015; UNFCCC 2015). It is noted that the reference period 1980-2010 is 0.6°C warmer than pre-industrial levels (IPCC, 2013), implying that the 1.5°C and 2°C warming targets are actually corresponding to global warming of 0.9°C and 1.4°C above the reference period, respectively (Lissner and Fischer 2016). By this, most of the historical training climate lies inside future temperature and precipitation ranges (Supplementary Figure S1).

Specifically, the gridded temperature and precipitation corresponding to the 1.5°C and 2°C warming world relative to pre-industrial era are first extracted from climate model simulations using the time-slice method (Schewe *et al* 2014; Leng *et al* 2015; Gosling *et al* 2016; Lissner and Fischer 2016), and aggregated to the county scale with weights based on the harvest area. Changes in temperature and precipitation relative to the reference period are then calculated for each county and used for perturbing the observed climate time series to construct future climate scenarios. Future maize yields simulated by ML driven with the climate scenarios are compared with yields in the reference period for evaluating the changes in yield average and low yields. The above processes are repeated for each climate model, based on which the multi-model ensemble mean is used for analysis with the inter-model spread indicating the uncertainty from climate models.

## 3 Results

**Figure 1:**
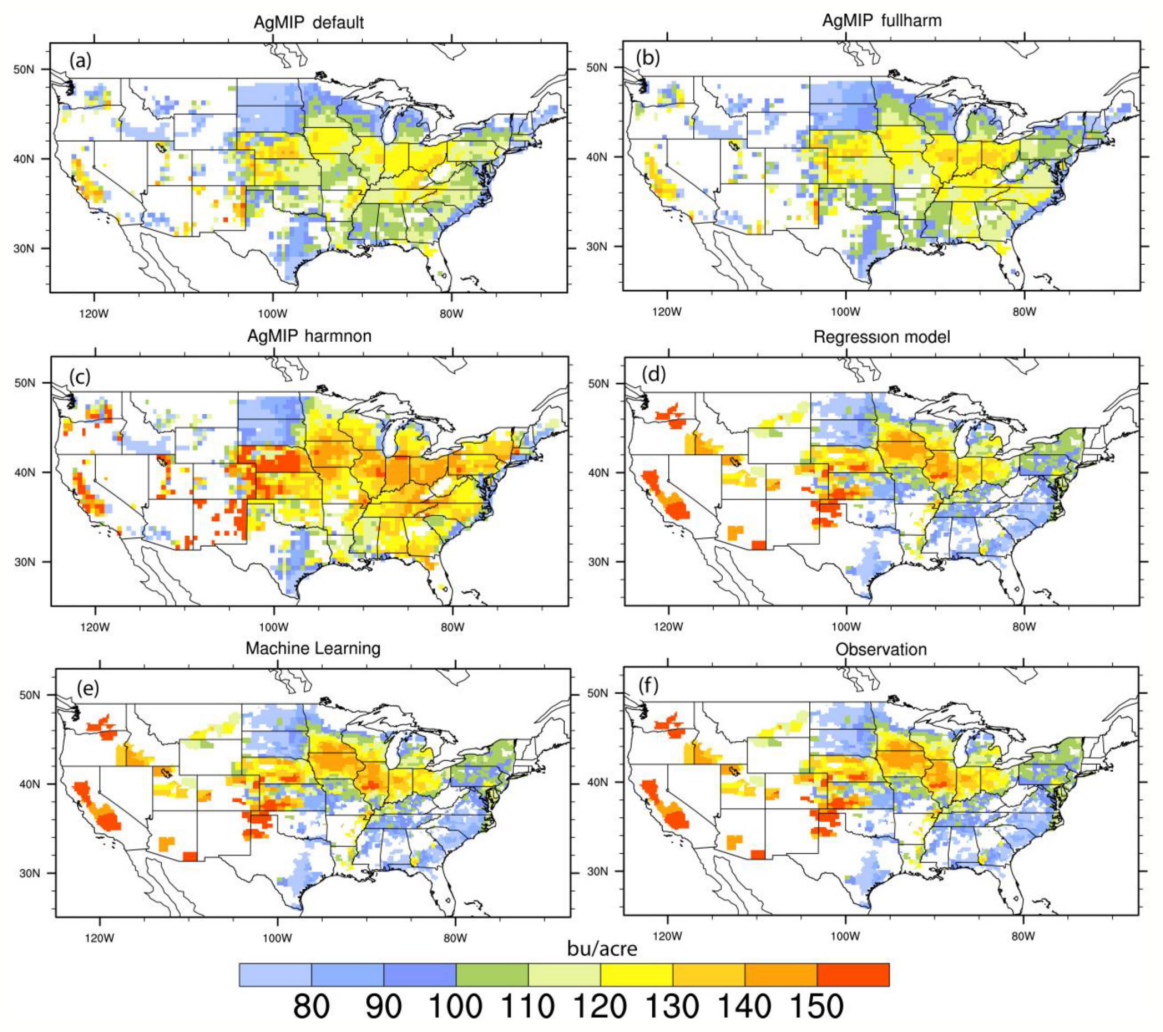
Spatial distribution of historical mean maize yield (bu/acre) for each county across the country. (a), (b) and (c) are for the ensemble mean of AgMIP model simulations under the default, fullharm and harmnon settings, respectively. (d) and (e) are based on regression model and machine learning, respectively. (f) is for census-based observation.

Figure 1 shows the long-term mean county-level maize yield simulated by the regression model, ML algorithm (i.e. Random Forests) and AgMIP models under different settings against observations for the period 1980-2010. It is evident that process-based models under the default settings show large bias in yield simulations, not only in the spatial distribution pattern but also in the magnitude of long-term mean yields. Such bias remains substantial when models are run even under the fullharm settings (i.e. assuming no limits of water and nutrient applications). When fed with observed management parameters (e.g. growing season, fertilizer input) in the harmonized settings (Elliott *et al* 2015), simulated yields by process-based models tend to match closer with observations in most of the country, though overestimation still exists in southern parts of the Mid-west corn belt and much of southeastern US. The varying performance under different management settings suggest that representation of agricultural management is an important uncertainty source for crop yield simulations.

Compared to process-based crop models, both regression and ML models show better skills in reproducing the observed spatial distribution patterns of maize yield across the country due to the trend (Supplementary Figure S2), though they are driven by only three predictors (i.e. temperature, precipitation and time). Similar findings are obtained when looking at the extremes of maize yield, with regression and ML models better capturing the observed spatial patterns of 10^th^ percentile yield (Supplementary Figure S3). However, caution should be exercised when interpreting the results from the above inter-comparisons, because both regression model and ML have been trained against observations, while limited calibration is done in the simulations of process-based crop models (Elliott *et al* 2015). Nevertheless, it is still important to recognize the promising skills of the regression model and ML algorithm for yield simulations, especially given that substantial inputs and computer costs are required for driving process-based models.

**Figure 2:**
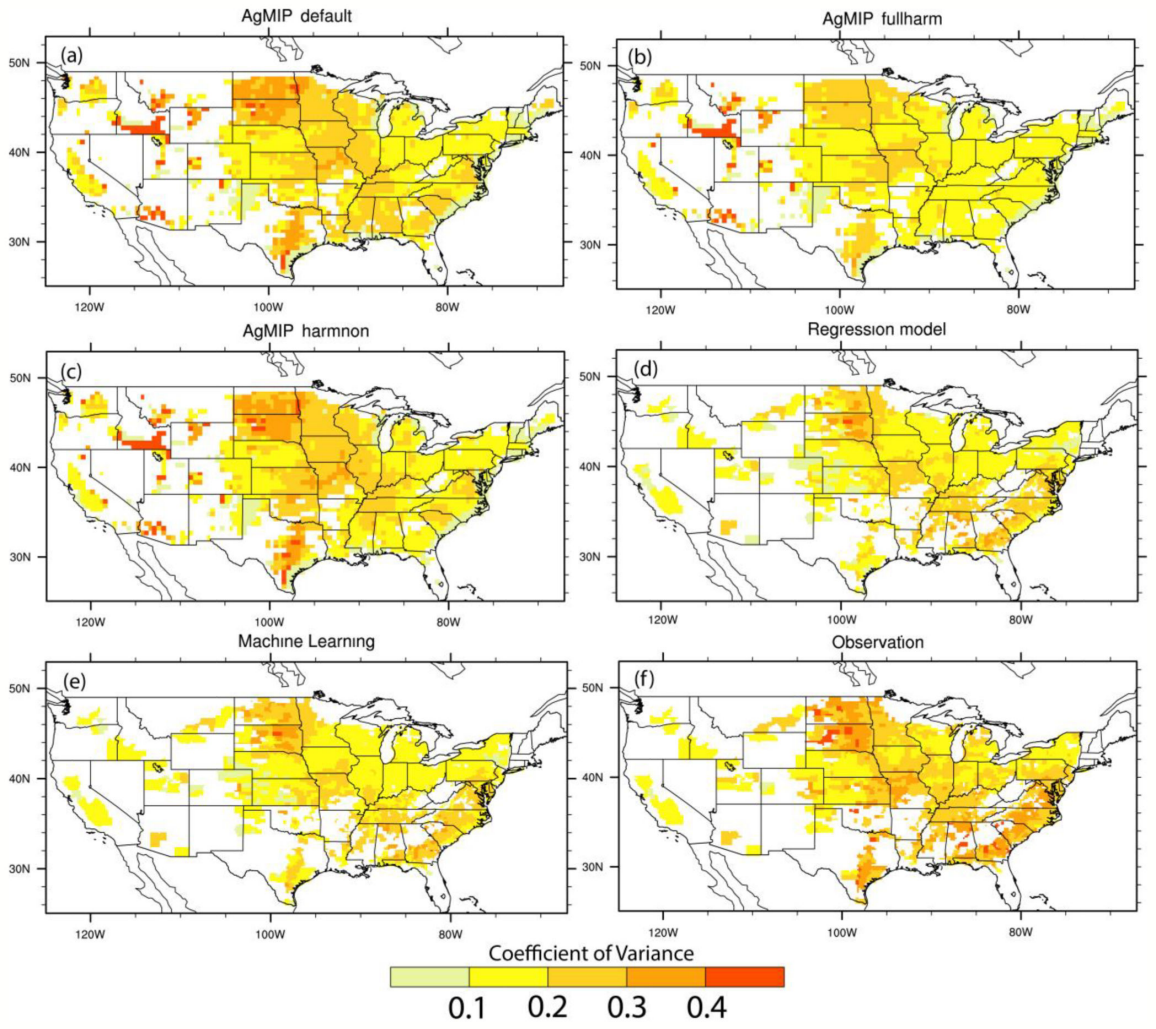
Spatial distribution of historical maize yield variability for each county across the country. Yield variability is measured by the coefficient of variance (CV), which is defined as the standard deviation of annual yields divided by the long-term mean during the period 1980-2010. High values of CV indicate large year-to-year variability relative to its long-term mean, and vice versa.

As for the year-to-year variation of maize yield, all models show similar spatial distribution patterns with larger values of coefficient of variance (CV) in the northern and southeastern parts of the country (Figure 2). Compared to observations, both regression model and ML tend to underestimate the magnitude of historical yield variability, which may be induced to some extent by the assumption of fixed growing season, though the bias is relatively small (supplementary Figure S4).. Process-based AgMIP crop models show mixed performances and the ensemble mean gives a overestimation in much of western country, which is mainly due to the underestimation of yield averages (Figure 1a-c). Similar to the simulation of yield averages, the performance of process-based models depends on the settings of agricultural management, and the sign of bias even differs among the different settings in the Midwest corn belt (Supplementary Figure S4a-c). Overall, process-based crop models tend to perform better in simulating the year-to-year variability of maize yield than its long-term averages. The results have great implications for enhancing the agricultural resilience to environmental risks, since it is the year-to-year variability that dominants the stability of food supply and food security.

**Figure 3:**
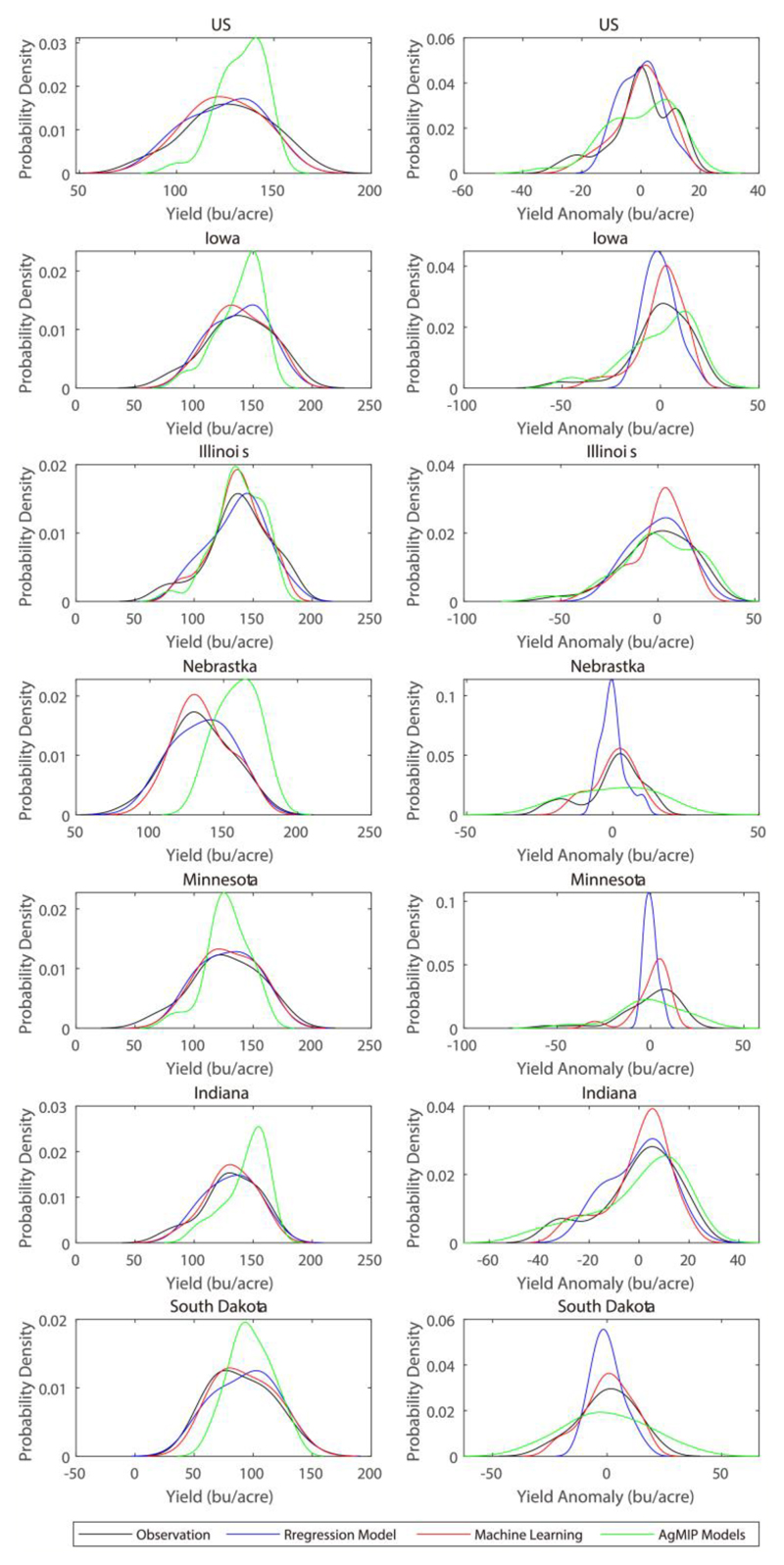
Probability density distribution (PDF) of maize yield from observations and simulations by regression model, machine learning and AgMIP models under the harmnon setting for the country as a whole and top six maize producing states. The left column shows the PDF of the absolute maize yield, while the right column is based on yield anomaly after excluding its change trend. Annual gridded yields are aggregated to the country and state scales with weights determined by the harvest area, based on which the PDFs are fitted.

Figure 3 shows the probability density distribution (PDF) of maize yield from observations and simulations by regression model, ML and process-based crop models under the harmnon setting. For the country as a whole, process-based models show large bias in simulating historical maize yield distributions, not only in the peak but also in the PDF tails. Instead, both regression model and ML well capture the observed shape of the probability density curve, but show slight bias in the peak of PDF. Specifically, the regression model tends to shift the peak of yield PDF to the right of observations, while ML gives an estimate shifting to the left. As for yield anomaly (after excluding the change trends), process-based models have largely underestimated the peak value of yield PDF. This suggests that the impacts of climate variations on yields may have been underestimated in the process-based models, since yield anomaly is mainly driven by climate variations. The regression model captures well the observed peak value of yield PDF, but showing substantial bias in the negative tail of yield distribution. ML outperforms both regression model and process-based models not only in the peak but also in the tails of yield PDF, demonstrating the value of using ML for analysis of the yield variability and the risks of extreme low yields. Similar findings are observed for the top 6 maize producing states, but with regression model showing worse skill in reproducing the PDF of yield anomaly in Nebraska, Indiana and South Dakota. Overall, ML exhibits the best skill in simulating the PDF of maize yield and its anomaly, followed by the regression model and process-based models.

**Figure 4:**
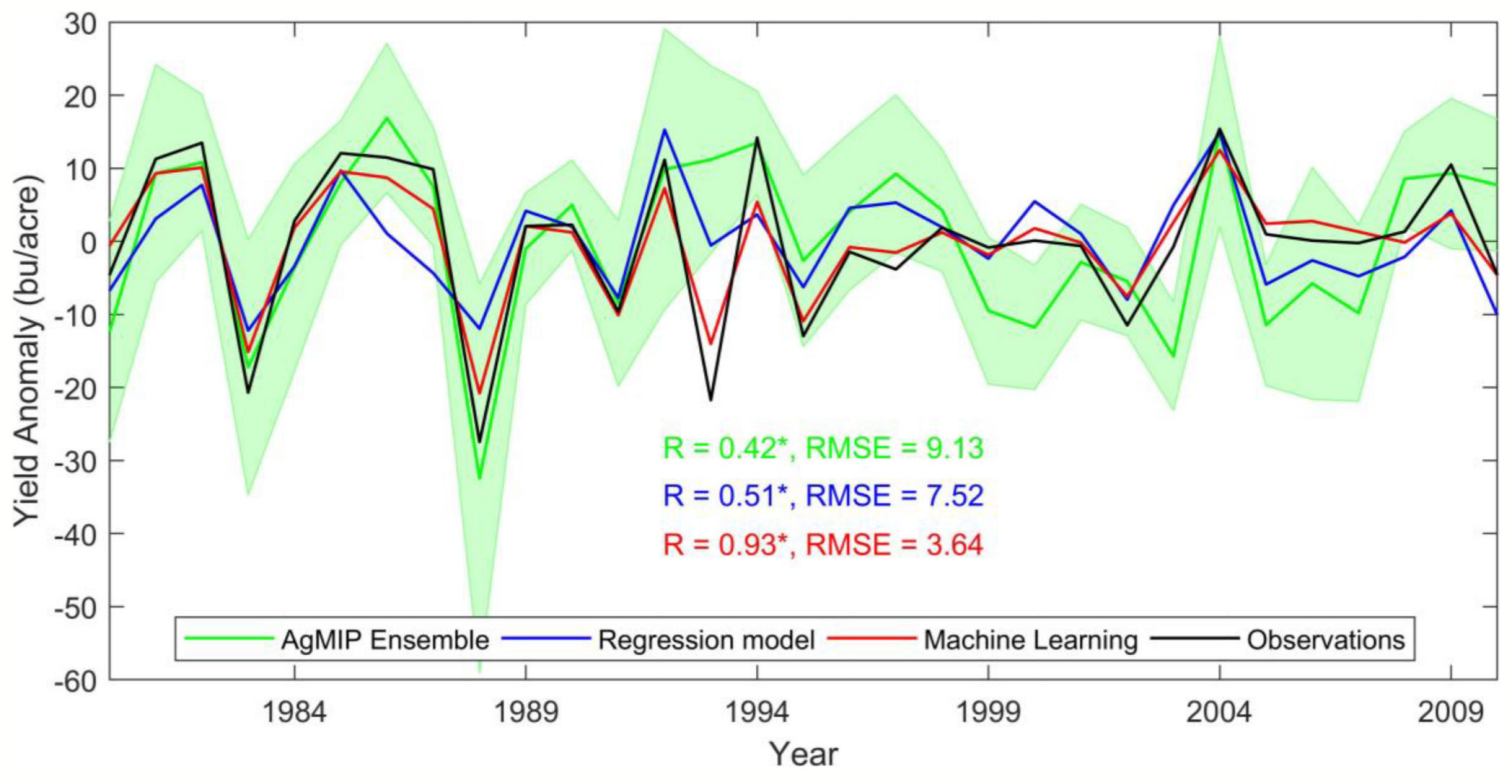
Temporal changes in historical maize yield anomaly from observations (black line) and simulations by AgMIP model under the harmnon setting (green line), regression model (blue line), and machine learning (red line). The shaded green area indicates the spread among the AgMIP models, while the solid green line represents the multi-model ensemble mean. The correlation coefficient (R) and rooted mean square error (RMSE) between simulations and observations are shown.

To assess the portion of yield variability that is contributed by climate variations, we perform ML and regression simulations driven by climate variables only (see method section 2.3). Both climate variables and yields are detrended, before they are used for fitting the models. Overall, our analysis indicates that climate variations can explain more than 40% of year-to-year variability of maize yield for the country as a whole (Figure 4). However, such estimates depend on the models that link yield variability to climate variations. Specifically, process-based models give the smallest estimates, with only 42% of yield variability explained by the inter-annual climate variations. Regression models which empirically fit the linear climate-yield relation indicates that 51% of US maize yield variability is induced by climate variations. Based on the ML algorithm, climate variation can explain 93% of historical yield variability, suggesting that previous estimates of climate impacts on yield anomaly may have been largely underestimated.

**Figure 5:**
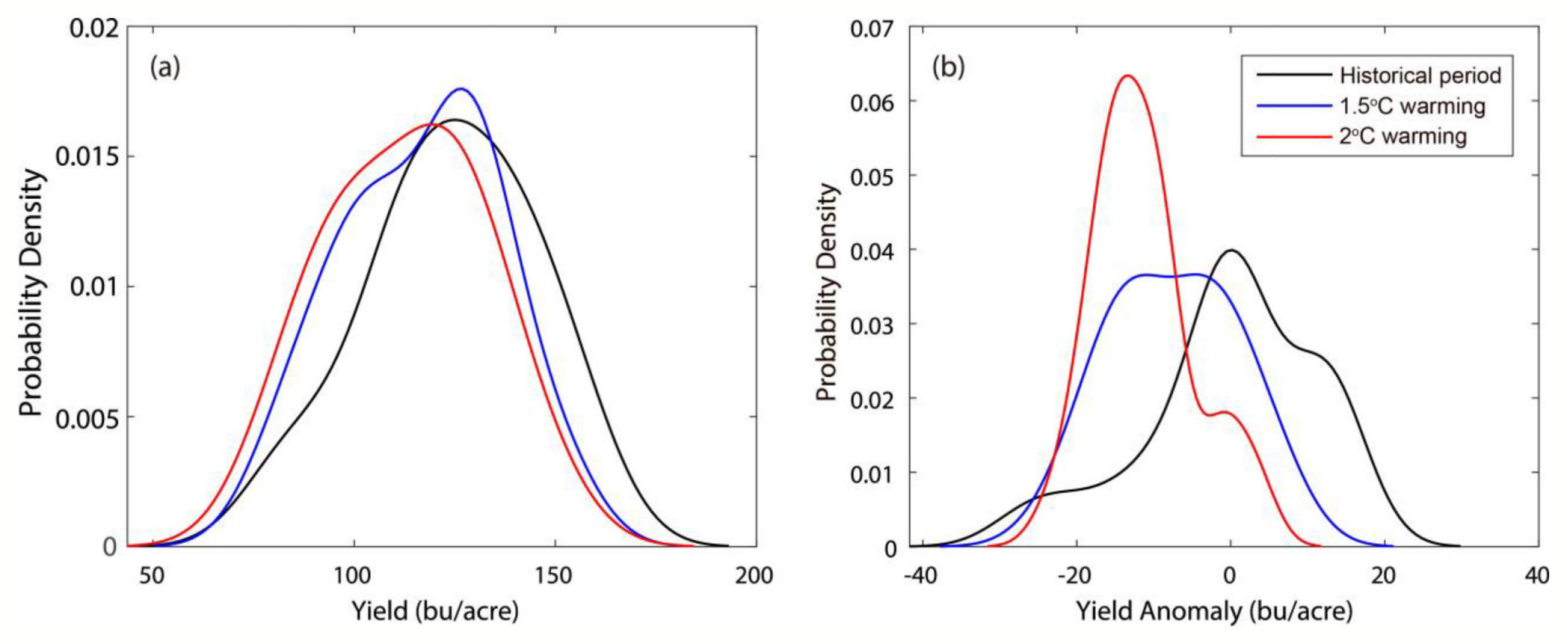
Changes in the probability density distribution (PDF) of maize yield in the 1.5°C (blue line) and 2°C (red line) warming scenarios relative to the historical period (black line). (a) is based on country-level absolute yields, while (b) shows the PDFs of yield anomalies (after excluding the change trends).

**Figure 6:**
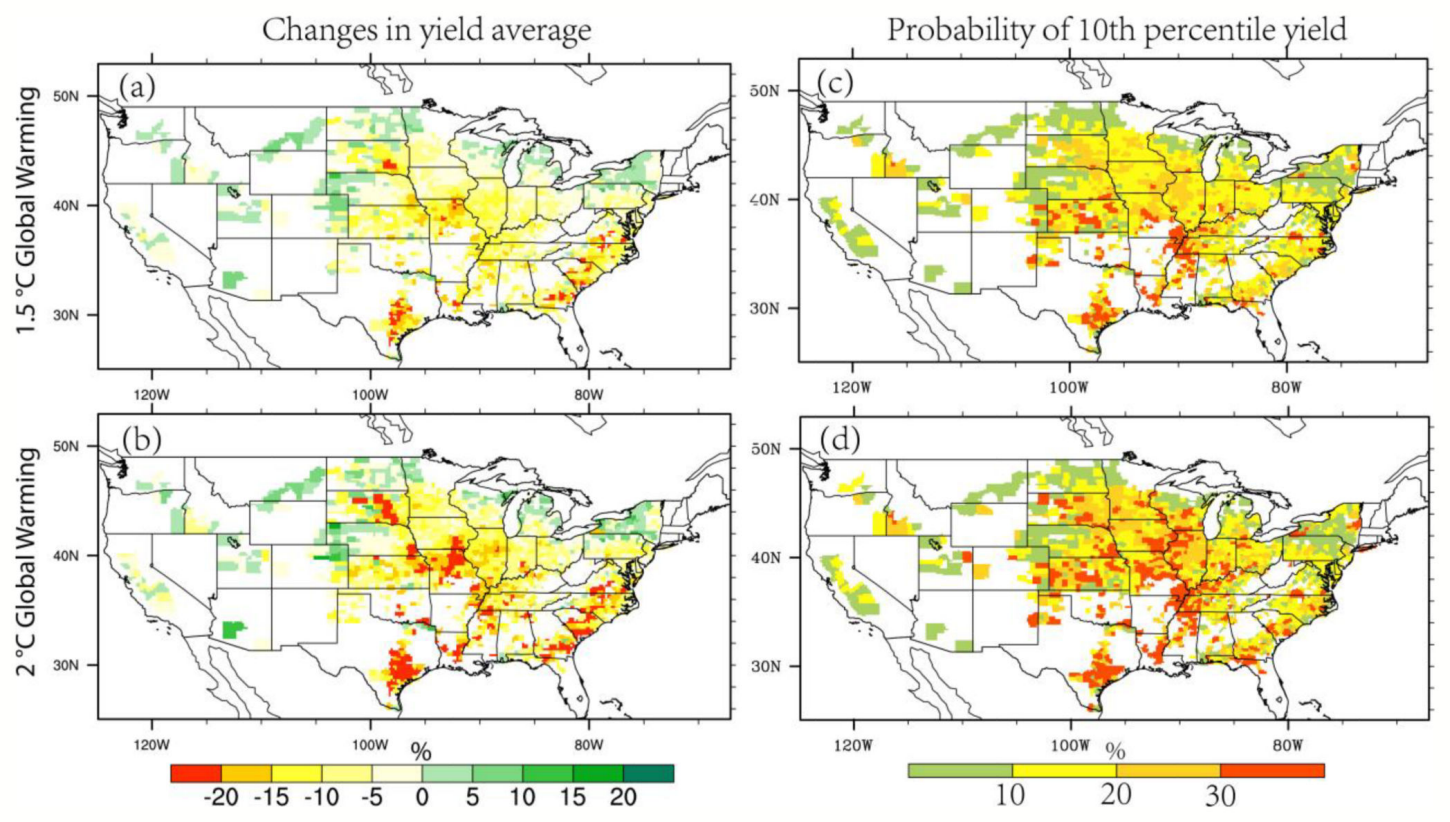
Spatial distribution of maize yield changes in the 1.5°C and 2°C global warming scenarios relative to the historical period. (a) and (b) are for the changes in yield averages, while (c) and (d) show the occurrence probability of historical 10^th^ percentile yield in the warming scenarios.

Given the promising performance of ML, we simulate US maize yield changes under the 1.5°C and 2°C warming scenarios by driving the ML model with climate model projections (see method section 2.4). As shown in Figure 5, a shift of yield probability distribution curve to the left is observed, which is more pronounced under the 2°C warming (by ~2050s) than the 1.5°C warming (by ~2040s). This suggests that US maize yield is likely to decrease in the future, especially under the 2°C warming world. On average, yield is projected to decrease by 9.0% and 13.5% under the 1.5°C and 2°C warming scenarios, receptively, which are broadly consistent with previous estimations (Schlenker and Roberts 2009; Butler and Huybers 2013). Spatially, a decrease in maize yield is predicted for most US counties under the 1.5°C and 2°C global warming scenarios (Figure 6a,b). The most severe yield loss is projected in the hottest states in the central and southeastern US, while the northern US where temperature is lower than the optimal level for crop growth is likely to benefit from future warming climate. The magnitude of maize yield loss tends to grow progressively in much of the country with increase in temperature, especially in the Southeastern growing areas. Using the ML model, we predict that maize yield could decrease by more than 15% in most of central and southeastern part of the country under the 2°C global warming. Constraining global temperature rise to 1.5°C would lead to smaller yield decrease in these hot-spot regions. The results have great implications for informing targeted adaptation measures, through identifying the hotspot regions where future climate change impacts are most severe.

Figure 6 further shows that extreme low yield defined as the historical 10^th^ percentile yield will occur more frequently in the future. This change in the frequency of extremely low yields potentially has great implications for food security, given that an extreme yield anomaly would typically cause larger impacts on the price and markets. Spatially, most of the maize growing areas will experience a substantial increase in the probability of extreme low yields, while a slight decrease is projected in the northern parts of the country due to the benefits of climate warming (Figure 6c,d). In Texas, Kansas, Missouri and Southeastern parts of the country, the ML model projects that the probability of extreme yield anomalies would even increase from 10% up to 30% under 2°C warming.

## 4 Discussions

Understanding climate impacts on crop yields is critical for adaptation and mitigations. Previous assessment relies on regression models or process-based models. This study demonstrates the potential utilization of ML for assessing climate impacts on crop yields, and a comprehensive comparison among process-based models, regression and ML is conducted, which is extremely valuable in improving our understanding of the implications of alternative methodological approaches. However, there are several limitations that should be acknowledged when interpreting the results of this study.

First, we choose June-July-August (JJA) as the major growing season for US maize, following the literature (Lobell *et al* 2013). Sacks and Kucharik (2011) reported that US maize growing period has increased by 12 days for the period 1981-2005. Since our study is based on the seasonal mean climate (i.e. average of growing season climate), a shift of few days would not affect the overall conclusions. Indeed, Lobell and Field (2007) showed that the particular choice of growing season did not greatly affect their estimation of climate impacts on crop yields. Schlenker and Roberts (2009) confirmed this by conducting a series of sensitivity analysis and found qualitatively similar results for eight alternative specifications of US maize growing season. Therefore, we choose the JJA period as the major growing season for US maize, and a fixed growing season has been widely adopted in many previous works (Lobell and Asner 2003; Mishra and Cherkauer 2010; Zipper *et al* 2016; Mistry *et al* 2017).

Second, there are several other climatic factors (e.g. vapor pressure deficit) that are neglected in this study. Given limitations in both statistical and process-based models, the goal of this paper is not to present an accurate yield prediction, but rather to compare and understand more fully their different level of performances. Therefore, the commonly adopted temperature and precipitation are selected, since they are the major climatic predictors of yields and their effects are represented in both statistical and process-based models. We show that even conditioned on temperature and precipitation only, ML and regression model tend to show promising performances. Further sensitivity analysis could be conducted to assess the benefits of including other climatic variables, though we suspect that this would be relatively small compared to precipitation and temperature.

Third, process-based models are not fully calibrated, while regression model and ML are trained against observations. It is well recognized that fully calibrating the process-based models is non-trivial, given the large numbers of uncertain parameters and lack of site-specific data. Especially, calibration of 11 process models would require tremendous efforts and coordination of different crop model development groups. To demonstrate the importance of model calibrations, we use and compare the simulations under three different settings (i.e. the default, fullharm and harmnon), which vary in the treatment of certain parameters. It is noted that some of the parameters related to managements are harmonized under the fullharm and harmnon settings, though they are not fully calibrated. Such a treatment does lead to better performance in yield simulations, especially under the harmnon settings (see the results section).

Fourth, our estimates of future yield changes are conducted without considering adaptations (Butler and Huybers 2013; Challinor *et al* 2014a), CO2 fertilization effects (McGrath and Lobell 2013; Deryng *et al* 2016), technology change (Najafi *et al* 2018) and other important factors such as pests, weeds and nutrients (Tubiello *et al* 2007). For example, the negative temperature impacts on crop yields could be reduced through soil mulching (Qin *et al* 2015), conservation tillage (Karlen *et al* 2013) and multiple cropping (Seifert and Lobell 2015). Recent observation-based studies also showed that irrigation can well buffer and mitigate the negative climate impacts on US maize yield (Troy *et al* 2015; Leng 2017a), though irrigation may be constrained by water availability, in particular in more severe drought scenarios.

## 5 Conclusions

While predicting crop yield response to climate change and variability has received a lot of attentions, it is not well explored in terms of yield variability and extremes. Here, we use the process-based models and traditional regression model as well an ML algorithm to investigate climate impacts on the average, variability and extremes of crop yields, using US maize yield as an example. Compared to process-based models, both regression model and ML show better skills in simulating yield averages, but still tend to slightly underestimate the year-to-year variability of yields in major production regions. Whilst the ensemble mean of process-based models can well reproduce the observed patterns of maize yield variability, there are substantial biases in predictions of yield averages. Notably, observed yield anomalies (after excluding the trend term) are almost completely reproduced by ML (R2=0.93), pointing to the promising skill of ML in assessing yield anomalies induced by climate variations. This also suggests that climate variation may have played a more dominant role in yield anomaly than previous estimations based on regression and process-based crop models, which reproduce less than half of observed yield anomalies.

We also found that the performance of process-based models largely depends on the parameterizations of agricultural managements (e.g. planting date and irrigation). When fed with harmonized management parameters, current state-of-art process-based models show improved skills in major production regions, though substantial bias remains in southeastern US. This suggests that representation of agricultural managements is an importance uncertainty source in process-based crop models. Nonetheless, the empirical (regression and ML) methods do not explicitly use parameterization of agricultural management, but are still able to better represent yield variations in many cases, conditioned only on climate variables.

Based on the historically trained ML model, US maize yield is projected to decrease by 9.0% and 13.5% under the 1.5°C (by ~2040s) and 2°C (by ~2050s) global warming scenarios, respectively. However, such estimates cannot be interpreted as predictions, as many factors influencing yields and the potential adaptation effects are not included. Given the ML algorthim’s improved capability for predicting yield variations, we used it to examine the future change in the frequency of extreme low yields. Using the yield that is currently at the 10^th^ percentile in the yield distribution, we predicted how frequently (i.e. at what percentile of the yield distribution) this would occur in future. Nationally we found that yields would be less than or equal to this benchmark low yield in 19% and 25% of years in 1.5°C and 2°C global warming scenarios, with potentially significant implications for food supply, prices and trade.

Understanding crop yield response to climate change and variability is fundamental for mitigating agricultural risks within the context of global warming. Pervious assessments of climate impacts are mainly aided with either process-based models or regression models. Here, we show that machine-learning algorithm is powerful for predicting crop yields, even though the model is conditioned on only two climate variables (i.e. temperature and precipitation). Process-based models will continue to be invaluable tools for projecting long term trends and the impacts of future changes, but could be supplemented by machine learning to downscale temporal and spatial patterns of yield average, variability and extremes. This will be particularly important for analysis of the risk of low yields in climatic extremes.

## Supplementary Material

Supplementary
