# Supplementary material for: Predicting spatial and temporal variability in crop yields: an inter-comparison of machine learning, regression and process-based models
Source: Environ Res Lett. Author manuscript; Available in PMC 2020 May 11. (PMC7212054; doi:10.1088/1748-9326/ab7b24)
Supplement: Supplementary [file EMS86278-supplement-Supplementary_.pdf]

Supporting Information for

Predicting spatial and temporal variability in crop yields: an inter-comparison of machine learning, regression and process-based models

Guoyong Leng[1,2,*] and Jim W. Hall[2]

[1]Key Laboratory of Water Cycle and Related Land Surface Processes, Institute of Geographic Sciences and Natural Resources Research, Chinese Academy of Sciences, Beijing 100101, China
[2]Environmental Change Institute, University of Oxford, Oxford OX1 3QY, UK

Contents of this file

Tables S1 to S2

Figures S1 to S4

---

*Corresponding author address: Guoyong Leng, Institute of Geographic Sciences and Natural Resources Research, Chinese Academy of Sciences, Beijing 100101, China
E-mail: lenggy@igsnrr.ac.cn

Table S1 Description of crop models used in this study

Crop Model	Model Type	Calibration	Calibrated Parameters	Key literature
CGMS-WOFOST	Spatially distributed site-based process model (based on WOFOST)	Site-specfic	NA	(de Wit and Van Diepen, 2008)
CLM-Crop	Global ecosystem model	Uncalibrated	NA	(Drewniak et al., 2013)
GEPIC	Site-based process model (based on EPIC)	Site-specfic	fertilizer application rate, Potential harvest index	(Liu et al., 2007; Williams et al., 1983)
LPJ-GUESS	Global ecosystem model	Uncalibrated	NA	(Lindeskog et al., 2013)
LPJmL	Global ecosystem model	National	maximum LAI, harvest index, factor for scaling leaf-level photosynthesis to stand level	(Waha et al., 2012)
pAPSIM	Site-based process model	Site-specfic	NA	(Keating et al., 2003)
PEGASUS	Global ecosystem model	Global	radiation-use efficiency factor	(Deryng et al., 2016)
EPIC-IIASA	Site-based process model (based on EPIC)	Site-specfic and global	fertilizer application rate, Potential harvest index	(Izaurralde et al., 2006; Williams et al., 1983)
EPIC-Boku	Site-based process model (based on EPIC)	Site-specfic	NA	(Izaurralde et al., 2006; Williams et al., 1983)

ORCHIDEE-crop	Global ecosystem model	Uncalibrated	NA	(Wu et al., 2016)
pDSSAT	Site-based process model	Site-specfic	NA	(Jones et al., 2003)

Table S2 Decriptions of 5 GCMs used in this study and the 30-yr periods corresponding to 1.5°C and 2.0°C warming targets under RCP8.5 scenario.

Model name	Institute acronyms	Institute full name	1.5°C	2.0°C
GFDL-ESM2M	NOAA GFDL	NOAA Geophysical Fluid Dynamics Laboratory	2021-2050	2035-2064
HadGEM2-ES	MOHC (additional realizations by INPE)	Met Office Hadley Centre and Instituto Nacional de Pesquisas Espaciais	2001-2030	2011-2040
IPSL-CM5A-LR	IPSL	Institut Pierre-Simon Laplace	2008-2037	2010-2049
MIROC-ESM-CHEM	MIROC	Japan Agency for Marine-EarthScience and Technology, Atmosphere and Ocean ResearchInstitute (The University of Tokyo), andNational Institute for Environmental Studies	2004-2033	2014-2043
NorESM1-M	NCC	Norwegian Climate Centre	2013-2042	2026-2055

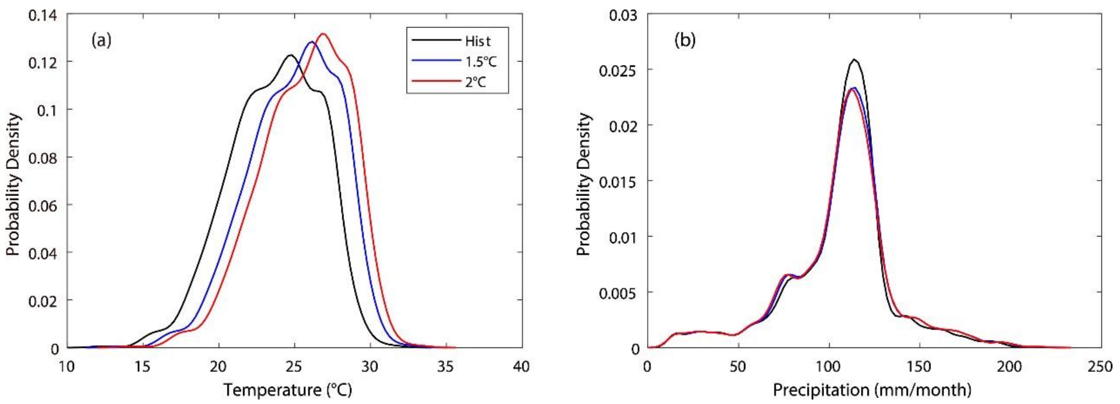

Figure S1 Probability density distribution (PDF) of growing season temperature (a) and precipitation (b) in the model training period (black line) and the 1.5°C (blue line) and 2°C (red line) warming scenarios. The PDFs are fitted based on county-level climates for the corresponding periods.

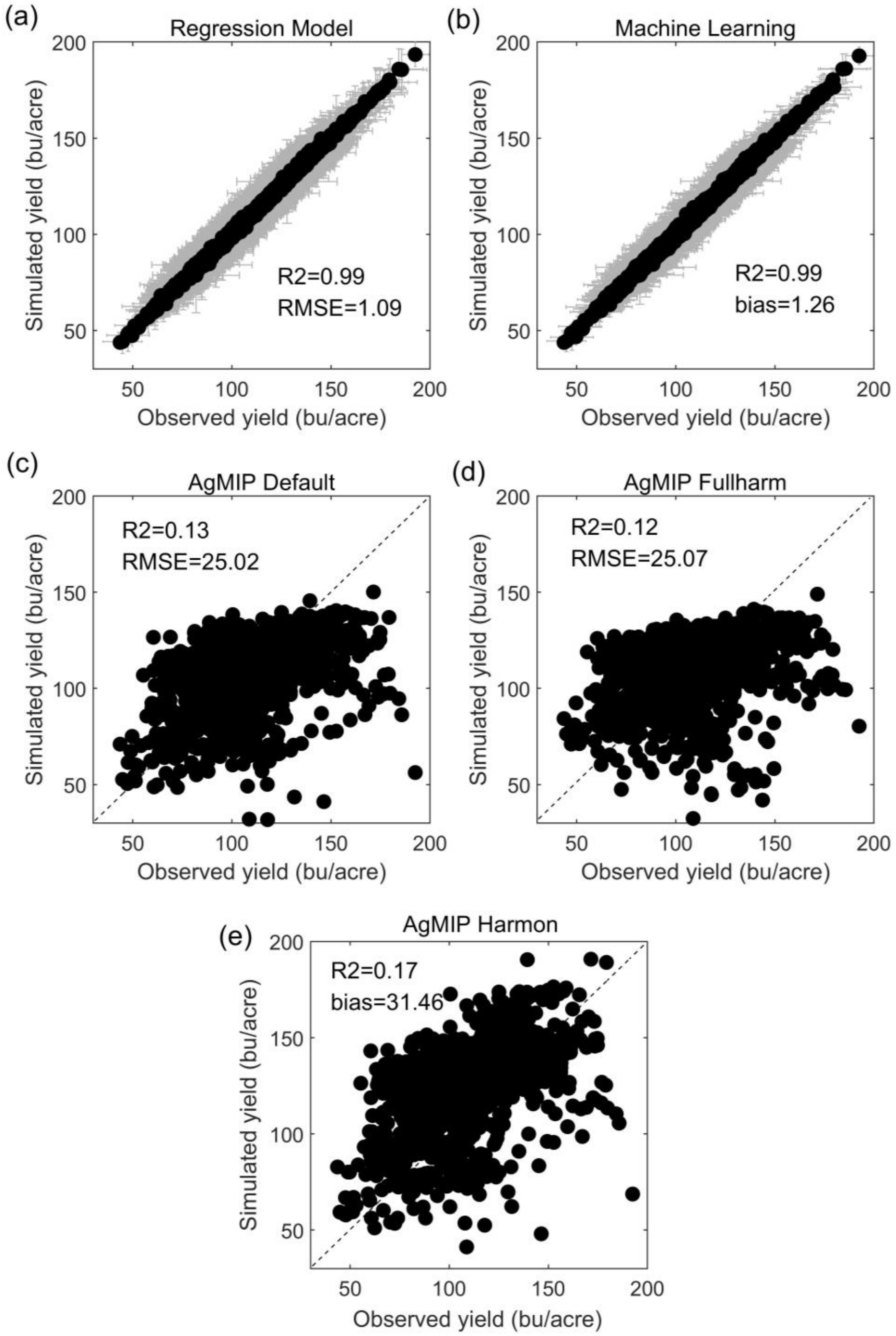

Figure S2 Comparison of simulated yields by (a) regression model, (b) machine learning and (c-e) AgMIP models under different management scenarios against observations from census data. 80% of datasets are used for calibrating both the regression and machine-learning models, with the remaining 20% data used for validating the model performance as shown here. Each dot indicates the long-term mean yield for a county. The error bar indicates the uncertainty from the limited samples used for training the models and is derived from 100 simulations through resampling techniques.

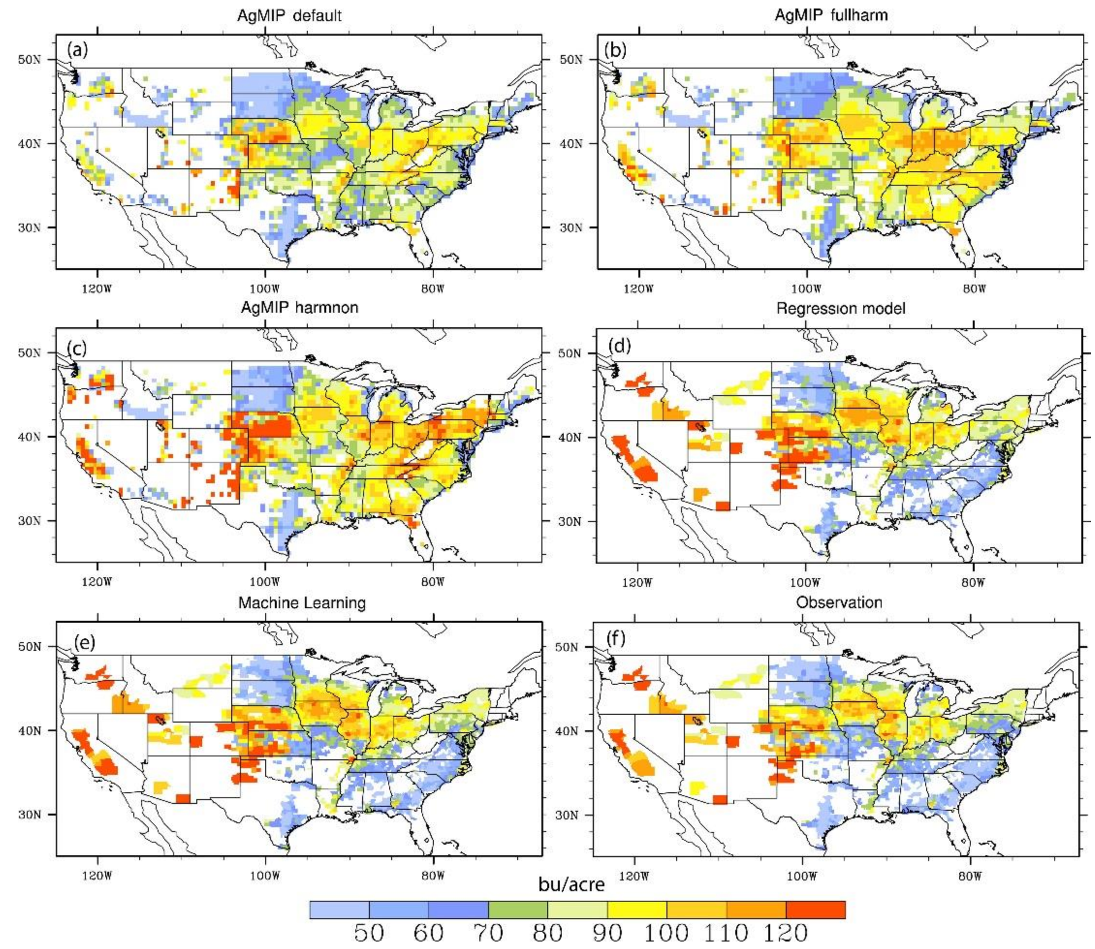

Figure S3 Spatial distribution of the 10th percentile yield during the 1980-2010 period from simulations by AgMIP models, regression model and machine learning as well as observations.

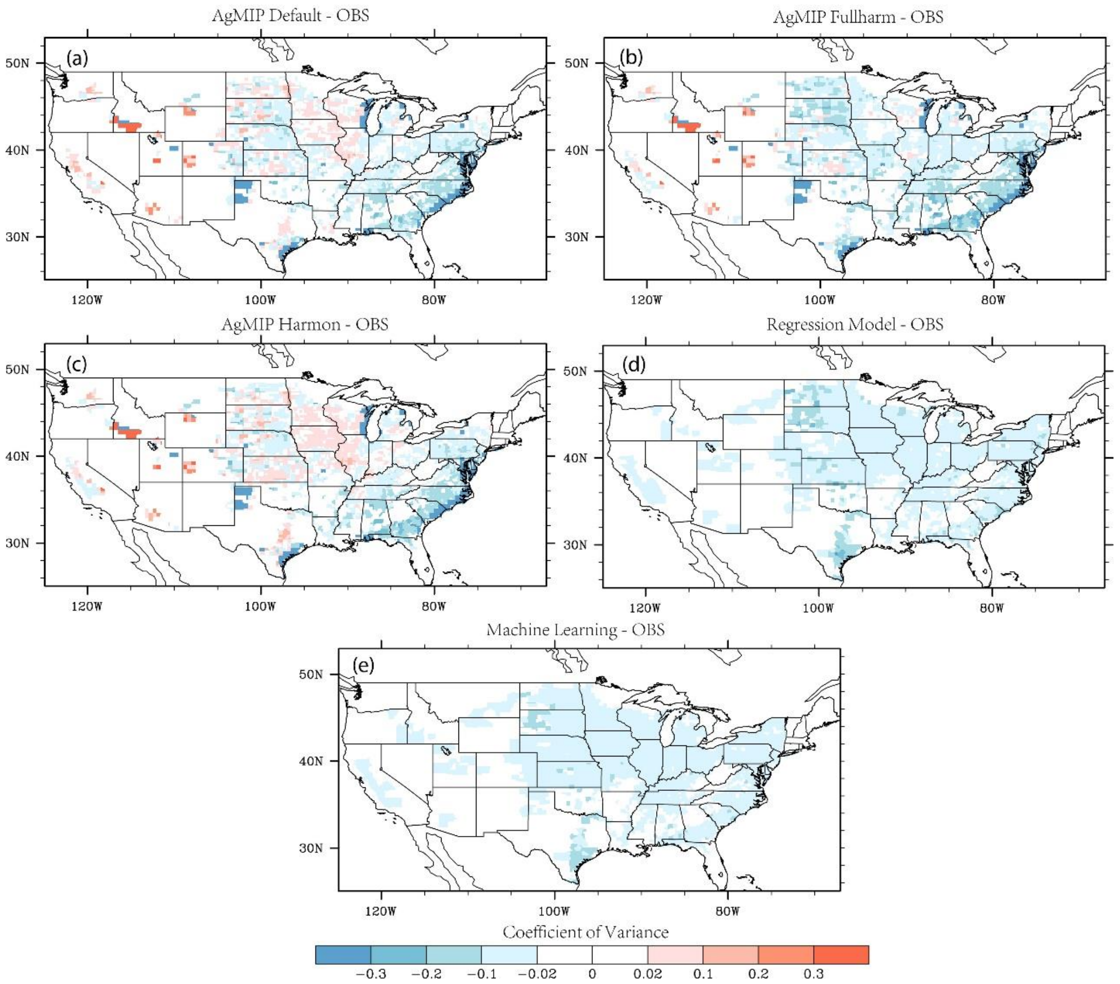

Figure S4 Difference in maize yield variability between simulations and observations for each county across the country. Yield variability is measured by the coefficient of variance (CV), which is defined as the standard deviation of annual yields divided by the long-term mean during the period 1980-2010.
